# The Orthopædic Department

**Published:** 1914-05-23

**Authors:** 


### THE ORTHOPÆDIC DEPARTMENT*

### VII. The Plaster Room (*continued*).

### MODERN METHODS IN PLASTER WORK.

In order to illustrate the method of employing plaster and its adjuvants, it is perhaps best to detail the *modus operandi* in a special case. Let us take, for instance, the two most common operations in modern orthopædic surgery, the treatment of club and flat feet. In the first, plaster is largely employed, while, in the second, celluloid is more advantageously used.

### Treating a Case of Club Foot.

The patient is a small child with equino varus, and it has been decided to do an achillotomy, with subsequent correction and plaster bandaging. The child is anæsthetised with ether, its foot placed on a "foot block," and the operator attempts to correct the deformity by patient and methodical redressment. If such correction under an anæsthetic is found to be impossible without severing the tendon Achilles, the ankle is cleaned with soap and water, followed by ether rubbing, and the little operation performed under aseptic conditions. A pad of sterile gauze is placed over the punctures, and the correction of the foot proceeded with. When the proper degree of correction has been obtained, the wound is dressed with collodium gauze, the foot is lightly sprinkled with sterilised chalk, and bandaged in the corrected, or slightly over-corrected, position. A couple of bandages "filled" with plaster are placed in cold water until they are thoroughly sodden; this takes on an average three to four minutes; when a large number of bandages are required an assistant is told off to place these bandages consecutively in the basin, and to hand them to the operator in succession in the order in which they have been placed in the water. This is necessary to prevent the plaster from becoming too liquid. The best kinds of bandages are covered with tissue paper, which need not be taken off before they are immersed in the water; if a number is made on the paper as each is put in, the saturation order is easily ascertained, and the consistency of the plaster as easily kept uniform. This is a slight point, but of some importance, since the best and firmest plaster is obtained by having all the bandages of a uniform consistency.

The assistant now takes the roll of bandage, still covered with its tissue-paper coating, in both hands and lightly squeezes the ends, so that all superfluous water is ejected from the roll which he then hands to the operator. The latter, an assistant holding the child's foot in the proper corrected position, then lightly and rapidly applies the plaster, never pulling at it, but allowing the folds to fall loosely in position as he makes the various turns. Starting a little above the ankle, he proceeds down to the instep and over the dorsum of the foot, reversing at the toes. A second bandage is put over the first; rarely a third is needed if the first two have been properly applied and the plaster is good. In a few minutes the plaster bandage is "set." Its surface is smoothed down—there is no necessity to smear plaster over the folds or to strengthen it in any way if the work has been properly done; all that is required is to trim the edges, and put over it a clean ordinary bandage. Such a bandage of plaster, when correctly applied, can be depended upon to last for several weeks, until, in fact, the cure is complete.

This is one of the easiest and simplest methods of applying a plaster bandage, and sufficiently explains the routine. More difficult plaster work is found in cases of congenital hip, and especially in applying plaster jackets or corsets. For a description of the technique in these more complicated cases the reader is referred to the German text-books on orthopædics, which give minute instruction on details. Applying plaster is an art which, unfortunately, the average student does not learn, unless he is fortunate enough to assist in an orthopædic department. Usually he contents himself with a lesson or two in making the antiquated splints, such as the Bavarian or Croft, useful enough in their way, but only so far as they reveal to him the great merits of plaster as an aid in surgery. Nearly every kind of fracture or deformity can be treated with an ordinary roll-plaster bandage, provided the operator knows how to apply his bandages. This knowledge can only be obtained by long practice and experience, which every practitioner should set himself to acquire, since plaster work ought to be much more extensively employed in private practice than it is at present.

It is well here to give a hint with regard to fracture cases and dislocations in which immobilisation in plaster has been adopted. When the plaster has been applied it ought to be a rule that the operator should test the correctness of his work by means of the *x*-ray screen. In cases of congenital hip reposition this is especially necessary, since during the manipulation of the limb in putting on the plaster the head of the femur may again have slipped out of the acetabulum. A thick plaster is almost impervious to the rays, and therefore a window must be made while the plaster is being applied, or it must afterwards be cut out before the splint has hardened. When this is properly done it is easy enough to get a view of the joint under the rays.

Plaster splints in hip cases and plaster jackets always require considerable trimming after they have been put on to make them wholly comfortable to the patient. When carefully adjusted, and when proper precautions have been taken to protect the underlying tissues by cotton-wool, cardboard, or aluminium strips, such trimming is comparatively easy. It is best done with a sharp knife, working from within outwards—*i.e.*, away from the patient's

* Previous articles appeared on November 1, 15, 29, and December 13, 1913, and January 24 and May 2, 1914.

skin—before the plaster has properly set. Once it has hardened the task is much more difficult, and the file or rasp must be employed.

Similar difficulties are encountered in removing plasters. Here, again, much assistance will be gained by making allowance for future difficulties in removing the plaster at the time when it is applied. For instance, by inserting a thin strip of aluminium beneath the line of subsequent section, the operator in removing the plaster can then cut boldly down in this strip of metal without danger of hurting the patient. In some books it is stated that old plasters can be removed by saturating the line of section with glacial acetic acid, which is said to soften the plaster; this is quite a fallacy. Such an aid may be of slight assistance in cases where the plaster contains a large amount of carbonate, which is attacked by the acid, but such plasters are generally very brittle and easily split when attacked by the knife. The ordinary firm sulphate plaster is not attacked by acetic acid, and must be removed by cutting instruments. There are many such instruments on the market, all more or less unsatisfactory. Perhaps the best is the Swedish scissors, made of hard Swedish steel; this cuts cleanly, and is very useful in moderately thick plasters. In others the knife and saw must be used. A very convenient way is to insert a long fret saw underneath the plaster and cut from below upwards, in the manner a Gigli saw is used. But in the best of circumstances the removal of a firm plaster splint is a laborious undertaking, especially when the plaster splint has to be used again. Where it can be destroyed piecemeal the best way of removing it is to immerse it in water for some time and then to unroll the bandages. Where the bandages have been applied "cleanly" this method is very good, but where plaster thickening has been used it is not always so easy to unroll the folds.

The most elaborate example of plaster bandage work is seen in the treatment of cases of spinal deformity, such as the forcible correction of kyphosis by Calot's method (now largely given up), and of scoliosis by the method of Abbott. In the former the patient is either suspended or treated on the table, and the adjustment of the bandages needs very careful consideration by the operator. The latter method, which is claimed to be the ideal treatment for certain cases of advanced scoliosis, and which, in capable hands, certainly gives most admirable results, is now very extensively employed, and has been well tried at Leeds, for instance. For a description of its technique we must again refer to the text-books, since it is too lengthy to be given here.

A word may be said as to the preparation of plaster bandages. Most surgeons prefer to buy their material ready made, and this has undoubtedly many advantages. For example, the ready-made bandages can usually be depended upon; they are made from tested plaster, and are of fairly uniform quality. Then, again, they are provided in air-tight metal or cardboard cases, so that each bandage is ready for use, and only needs immersion in water to be applied. There are so many kinds on the market that the surgeon has unlimited choice, and nearly every maker supplies more than one kind of bandage. But these ready-made bandages are by no means cheap; they average fivepence each for the larger sizes and twopence for the smaller. Self-made bandages are much cheaper, and can be made with comparatively little expenditure of time and trouble, provided the unit possesses a bandage-making machine. There are several patent machines which give excellent results, and an orthopædic department to be properly equipped should have one of these machines and prepare its own bandages. This is more economical in the long run; the machine soon pays its way, and old used bandages, sterilised, can be used as the basis for the new plaster bandages, thus effecting a considerable saving all round. Such machines are generally used in orthopædic departments abroad, and are stated to give satisfaction. The rollers can be adjusted for any size of bandage required, and the machine itself is easily worked by a boy or a nurse, and is capable of turning out several hundred bandages a day.

